# MiR26-5p inhibits pathological pulmonary microvascular angiogenesis via down-regulating WNT5A

**DOI:** 10.22038/IJBMS.2023.68856.15006

**Published:** 2023

**Authors:** Jie Chen, Feng Gao, Dan Li, Jinquan Wang

**Affiliations:** 1 Department of Anesthesiology, People’s Hospital of Chongqing Banan District, Chongqing 401320, China; 2 Department of Anesthesiology, The First Affiliated Hospital of Chongqing Medical and Pharmaceutical College, Chongqing 400038, China; 3 Southwest Hospital, Third Military Medical University, Chongqing 400038, China; 4 Department of Anesthesiology, The Ninth People’s Hospital of Chongqing, Chongqing 400700, China; # These authors contributed equally to this work

**Keywords:** Hepatopulmonary-syndrome (HPS), Migration, MiR26-5p, Proliferation, Pulmonary microvascular-endothelial cells, (PMVECs)Wingless-related - integration-site family, member 5A (WNT5A)

## Abstract

**Objective(s):**

Pathological micro angiogenesis is a key pathogenic factor in pulmonary diseases such as pulmonary hypertension and hepatopulmonary syndrome. More and more pieces of evidence show that excessive proliferation of pulmonary microvascular endothelial cells is the key event of pathological micro angiogenesis. The purpose of this research is to reveal the mechanism of miR26-5p regulating pulmonary microvascular hyperproliferation.

**Materials and Methods:**

Hepatopulmonary syndrome rat model was made by common bile duct ligation. HE and IHC staining were used for analysis of the pathology of the rat. CCK8, transwell, and wound healing assay were used to assess miR26-5p or target gene WNT5A functioned toward PMVECs. microRNA specific mimics and inhibitors were used for up/down-regulated miR26-5p expression in PMVECs. Recombinant lentivirus was used for overexpression/knockdown WNT5A expression in PMVECs. And the regulation relationship of miR26-5p and WNT5A was analyzed by dual-luciferase reporter assay.

**Results:**

qPCR showed that miR26-5p was significantly down-regulated in the course of HPS disease. Bioinformatics data showed that WNT5A was one of the potential key target genes of miR26-5p. Immunohistochemistry and qPCR analysis showed that WNT5A was largely expressed in pulmonary microvascular endothelial cells, in addition, this molecule was significantly up-regulated with the progression of the disease. Furthermore, dual luciferase reporter assay showed that miR26-5p could bind to WNT5A 3 ‘UTR region to inhibit WNT5A synthesis.

**Conclusion:**

The results suggested MiR26-5p negatively regulated PMVECs proliferation and migration by WNT5A expression. Overexpression of miR26-5p may be a potentially beneficial strategy for HPS therapy.

## Introduction

Hepatopulmonary syndrome (HPS) is a life-threatening disease, which develops from chronic liver disease and/or portal hypertension. Refractory hypoxemia is a combination of symptoms that can be as high as 47% and is an important cause of death in cirrhotic patients (1). At present, the most effective treatment for HPS in clinical work is liver transplantation, but facing the organ shortage crisis around the world, it is urgently needed. The pathogenesis of HPS should be further studied to provide an effective way for HPS prevention and treatment. Previous studies have widely believed that capillary dilation and arteriovenous malformation are the basis of pathological angiogenesis (2). However, in HPS, the alveolar-capillary pathologic angiogenesis regulatory mechanism is not clear.

Wingless-related integration site (WNT) family member 5A (WNT5A), is believed to regulate multiple ontogenies, physiological processes, and pathologic processes, including cell differentiation, proliferation, polarity, and migration (3, 4). It has been shown that during body embryogenesis, WNT5A is essential for it to execute its function in embryonic stem cell differentiation into endothelial cells (5). In human primary endothelial cells, WNT5A is highly expressed and stimulates cell proliferation, migration, and survival (6-9). WNT5A promotes the angiogenesis of melanomas by releasing exosomes containing pro-angiogenic proteins (10). However, the function of WNT5A and the transcriptional mechanism in regulating PMVECs angiogenesis remains unknown. Exploring the physiological function of WNT5A in pulmonary microvascular angiogenesis, which might be a key regulator mechanism of HPS, may provide a new way to prevent HPS.

MicroRNAs are a class of endogenous noncoding RNAs that are 20-24 nucleotides in length. It regulates the vast majority of gene expression and protein synthesis in many biological processes, including cell differentiation, proliferation, migration, body development, and tumorigenesis (11, 12). Generally, miRNAs regulate the target genes’ post-transcriptional expression by specifically inhibiting their translation (12, 13). Most reported miRNAs inhibit the protein synthesis process of the gene by binding to the 3’ UTR (13, 14). MiRNA associations have been reported to lead to translation inhibition, usually accompanied by extensive mRNAs degradation by a non-RNAi mechanism (15). Previous studies have identified that several miRNAs have a key role in the regulation of HPS progress, including miR-206, miR-9, and miR-199a-5p (16-18). These studies all have shown that these miRNAs regulate PASMC phenotypic regulation. However, microRNA regulation on PMVEC proliferation and angiogenesis in HPS should be paid close attention to. Studies also have shown that different miRNAs regulated the expression of WNT5A (19, 20). Here, we focus on miRNA regulating WNT5A to control the HPS pathogenesis.

In this study, we constructed an HPS rat model and investigated WNT5A expression in lung tissues and PMVECs. The putative microRNA of WNT5A was selected by bioinformatics analysis. Then, we identified that miR26-5p is an important negative regulator of WNT5A. In addition, the regulation of miR26-5p on PMVEC proliferation was analyzed by CCK-8 assay. We propose that miR26-5p/WNT5A operates as a crucial molecular in the regulation of HPS-related angiogenesis. This study serves as new insight into mRNA research with therapeutic potential for HPS.

## Materials and Methods

***HPS rat model***

This research was approved by the Animal Care Committee of the Third Military Medical University and in accordance with National Institutes of Health guidelines. Experimental SD Dawley rats (180~220 g) were provided by Laboratory Animal Center, Third Military Medical University. The HPS rat model was established by common bile duct ligation (CBDL) surgery (21, 22). Forty rats were selected for surgery. 15-40 mg/kg Pentobarbital Sodium was intraperitoneally injected into anesthetized rats. The CBDL group received CBDL surgery (n=30), and the control group received sham surgery (n=10); rats underwent common bile duct exposure instead of ligation. CBDL rat tissues were extracted at the end of the 1^st^, 3^re^, and 5^th^ weeks. Each stage used 10 rats for the experiment. 

***Cell culture***

As described in previous studies, we isolated pulmonary microvascular endothelial cells (PMVECs) from SD rat lungs for identification (23). Cells were cultured in endothelial basic culture-medium 2 (EBM-2) containing 10% fetal bovine serum at 37 ^°^C and with 5% humidity in a CO_2_ incubator.

***Bioinformatics analysis***

miRTarBase (http://mirtarbase.mbc.nctu.edu.tw) and Target scan human (http://www.targetscan.org/) were used to predict MiR26-5p potential targets.

***qRT-PCR***

TRIzol kit (Beyotime, Shanghai, China) was used to isolate rat lung tissue or PMVECs total RNA. And MMLV reverse transcriptase (Beyotime, Shanghai, China) was used to synthesize the first cDNA. All reagents are used by following the manufacturer’s instructions. cDNA synthesis was performed by adding miRNA-specific stem ring primers, oligonucleotides (dT), or random primers. SYBR®Green Quantitative PCR kit (Beyotime, Shanghai, China) was used for qRT-PCR. The relative gene mRNA levels were calculated by the 2-ΔΔCt method, and the GAPDH gene Ct values were used as internal reference to eliminate the differences between groups. The experiment was carried out at least three times. The primers of all experiments are shown in Table S1.

***Western blot***

Protein isolation and western blot were according to Yang *et al.* (23). In simple terms, rat lung tissues and cell total proteins were extracted, then isolated in 10% SDS PAGE gel after concentration determination, and then transferred to PVDF membrane. After BSA closure, primary antibody wnt5a (Abcam, USA) and anti-beta-actin (Abcam, USA) were diluted at 1:2000 and incubated overnight at 4 ^°^C. PBST was used to wash membranes three times. And then, the second antibody was incubated at 1:5000 dilution (Abcam, USA). An enhanced chemiluminescence system (Beyotime, Shanghai, China) was used to detect horseradish peroxidase-conjugated antibodies.

***Vector construction***

WNT5A gene (NM_022631) 3’UTR sequence of rat lung was amplified by PCR using specially designed primers. The upstream and downstream primers contained Xba I and BamH I restriction endonuclease sites, respectively. The target sequence was cloned into the pMD-19T vector (TaKaRa Biotech, Japan) for sequencing verification. The correct vector digest by Xba I and BamH I was verified, the target sequence obtained, and transferred to the luciferase reporter plasmid pGL3 vector (Promega, Madison, USA). The report carrier was named pGL3-LUC-WNT5A UTR. Based on the constructed pGL3-LUC-WNT5A UTR vector, a mutation vector of the miR26-5p binding site was constructed by site-specific mutagenesis. That is, the whole vector was amplified with special primers containing nucleic acid fragments of target site mutations, and the mutant vector obtained by amplification was transformed into receptive cells of *Escherichia coli*. Then, sequencing was carried out for verification, and endotoxin-free plasmid was isolated for the next cell transfection experiment. Table S1 lists all primers needed for the experiment.

***Cell Transfection and dual-luciferase reporter assay***

Cell transfection experiment was employed by analysis of regulatory between WNT5A and miR26-5p. It was performed using the dual luciferase reporting detection system (Promega, Madison, USA) in the GloMax-Multi detection System Photometer (Promega, Madison, USA). First, we synthesized MiR26-5p specific mimic, control mimic, inhibitor, and control inhibitor in Gene RIB Biotech (Guangzhou, China). Then, PMVECs cells were inoculated into 24 well plates, with 5×10^4^ cells per well. MiR26-5p mimics (50 nM) or MiR26-5p inhibitors (80 nM) were wrapped and transfected into PMVECs by Lipofectamine 3000 (Invitrogen, USA). During transfection, each wild-type or deletion vector was transfected into the cells at a dose of 500 ng, with the addition of 50 ng/well internal reference vector pRL-TK (Promega, USA). 24 hr after transfection, supernatant of cells was collected to detect luciferase activity, measured with Varioskan Flash Multimode Reader (Thermo Scientific, Waltham, USA). Each group of experiments was conducted independently 3 times (24).

***Immunochemistry***

The lung tissue was fixed with 10% formalin and then paraffin-embedded. The section thickness was 5 μm. The sections were dewaxed before staining, then closed with 5% serum for 2 hr and treated with primary antibody wnt5a (1:200, NO. ab227229, Abcam, UK) overnight, then incubated with horseradish peroxidase coupled secondary antibody (1:500, NO. ab6721, Abcam, UK) for 2 hr. The positive signal was detected using the DAB Horseradish Peroxidase Color Development Kit (Beyotime, Shanghai, China), and the nucleus of the tissue was then stained with hematoxylin. After the section was sealed, the sections were placed on a micrograph optical microscope (Olympus, Japan) for photo collection and quantified by Image-Pro Plus.

***Immunofluorescence***

First, treated PMVECs were fixed to sections using cell slides and blocked with 10% goat serum albumin for 2 hr. Then, sections were incubated with primary antibody wnt5a (1:500, Abcam, UK) overnight at 4 ^°^C. The primary antibody was removed, washed three times with PBST, and the cells were cultured in a 1:500 diluted fluorescent-labeled secondary antibody for incubator for 2 hr. After three washes in PBST, PMVECs were sealed with DAPI tablet solution. Finally, the sections were observed with confocal microscopy.

***Cell Counting Kit-8 assay***

PMVECs cells proliferation under different treatments was detected by Cell Count Kit 8 (CCK-8). To put it simply, the cells were laid out on a 96-well plate and treated. After completion, the cells were added to 10 μl CCK-8 solution (Beyotime, Shanghai, China) and incubated at 37 ^°^C for 2 hr. Next, the 450 nm absorbance was measured and analyzed by an enzyme marker Varioskan Flash Multimode Reader (Thermo Scientific, Waltham, USA).

***Wound healing assays***

First, PMVECs were inoculated into 24-well plates with 5.0×10^4 ^cells /well and starved with serum-free medium for 24 hr. Then the medium was replaced with 10% FBS. Cell wounds are created using the tip of a plastic suction head. After 0 hr, 6 hr, 12 hr, 24 hr, and 48 hr, the wound was photographed using a microscope. Finally, the cell coverage area (%) was quantitatively migrated by ImageJ. All experiments were repeated three times.

***Transwell assay***

Cells treated with the combinative virus or miR26-5p specific mimics/inhibitor were starved in serum-free medium for 8 hr. Seeds were then injected into each well (8 pm aperture, Corning, USA) across the upper chamber; 0.5 ml RPMI 1640 containing 10% FBS was added into the lower cavity. PMVECs were cultured for 24 hr in a CO_2_ incubator at 37 ^°^C and 5% CO_2_. Then, the cells in the upper part of the superior lumen were erased. After fixation with methanol and staining with 2% crystal violet for 10 min, 8 random visual fields were randomly selected under the microscope. All of the experiments were carried out three times.

***Statistical analysis***

Statistical analysis was done using SPSS 23.0 software (IBM, USA). Comparison between the two groups was performed by the *t*-test. One-way analysis of variance was adopted for the comparison of multiple samples. Data are shown as the mean±SD. *P*<0.05 represents statistically significant results.

## Results

***Pulmonary microvascular angiogenesis was found in the HPS rat lung***

**Figure 1:**
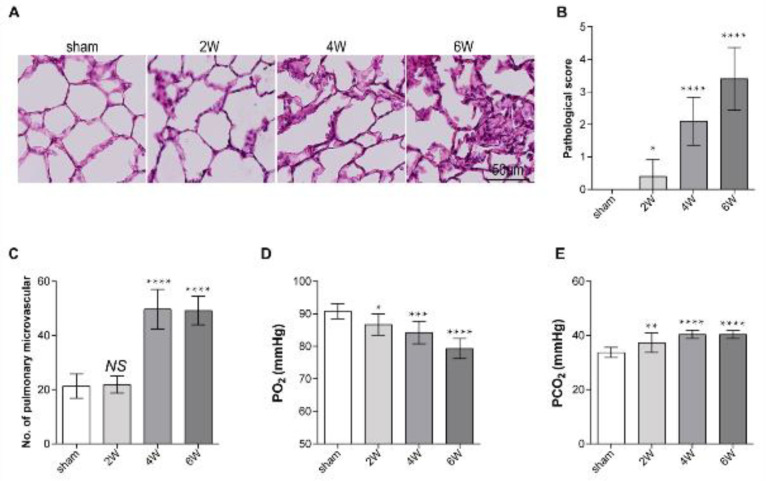
CBDL rat model induces pulmonary injury

First, the HPS rat model was established by common bile duct ligation (CBDL). The results showed that the alveolar epithelium was flattened and the alveoli were more unstable by H&E staining compared with the sham group (Figure 1A). CBDL rat lung injury scores are increased gradually with disease progression (Figure 1B). Then, pulmonary micro-vessel number in different visual fields in the same area was counted in each group, and it was found that compared with the sham operation group, pulmonary micro-vessels number in CBDL rats increased significantly. Then blood gas analysis was performed to assess the respiration of HPS rats. The experimental results showed that PO_2_ increased significantly and PCO_2_ decreased gradually with the progression of HPS disease (Figure 1D, E). This indicates that HPS model rats can better simulate the pathological state of HPS patients.

***WNT5A expression is induced in CBDL rat lung and promotes PMVECs proliferation and migration***

**Figure 2:**
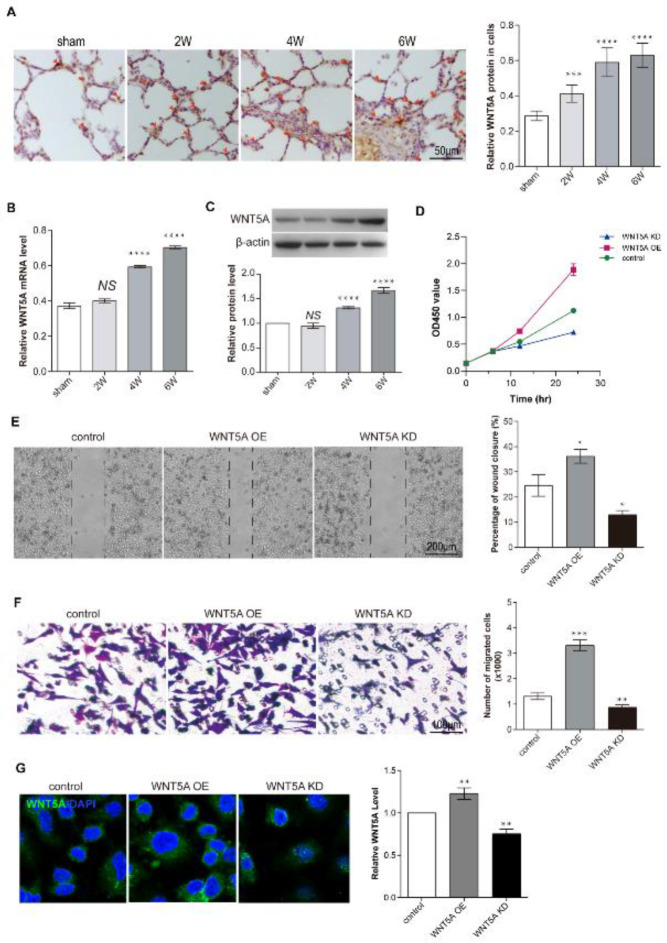
WNT5A expression is induced in CBDL rat lungs and promotes PMVEC proliferation and migration

To verify the function of WNT5A on pulmonary microvascular hyperplasia, we first analyzed the WNT5A expression pattern in CBDL rat lungs. Immunochemistry analysis and western blot demonstrated that WNT5A was weakly expressed in the sham group lungs. However, WNT5A expression in the HPS rat lungs increased gradually (Figure 2A, 2C). These results showed WNT5A still expressed low in the sham rat lungs. However, after CBDL surgery, the WNT5A mRNA gradually increased in the lung over time (Figure 2B).

Then, we used WNT5A-specific recombinant virus to over-express/knockdown WNT5A in PMVECs to analyze the effect on cell proliferation and migration. Immunofluorescence assay showed that WNT5A-specific overexpression recombinant virus can significantly induce WNT5A expression, and WNT5A-specific knockdown recombinant virus can significantly reduce WNT5A expression (Figure 2G). CCK8 assay showed that WNT5A overexpression cells have the fastest proliferation rate and also have the strongest migration ability, controlled by the control group and WNT5A knockdown group (Figure 2D, E, F). 

***MiR26-5p level is opposite to that of the WNT5A in the lung of rats***

Prediction of microRNA binding sites on WNT5A gene by bioinformatics analysis. We used miRTarBase and Target scan human to make predictions and found that there is a miR26-5p binding site on the WNT5A 3’UTR sequence (Table 1). We hypothesize that miR26-5p has an important role in the IPVD of HPS by modulating WNT5A expression.

**Figure 3:**
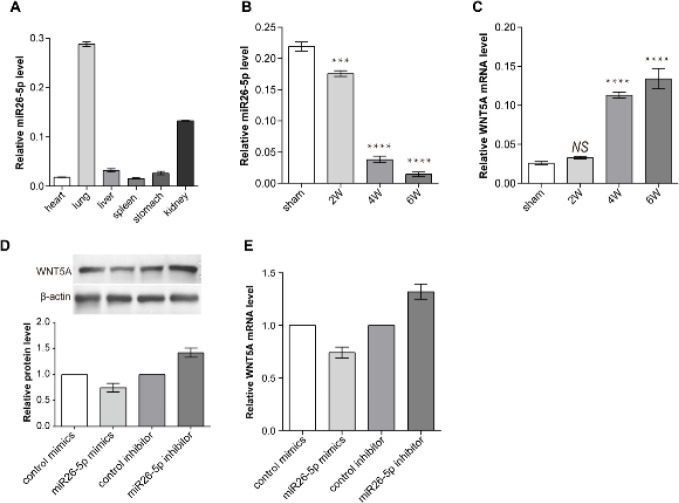
Expression trend of miR26-5p is opposite that of WNT5A in the CBDL rat and PMVECs

**Figure 4:**
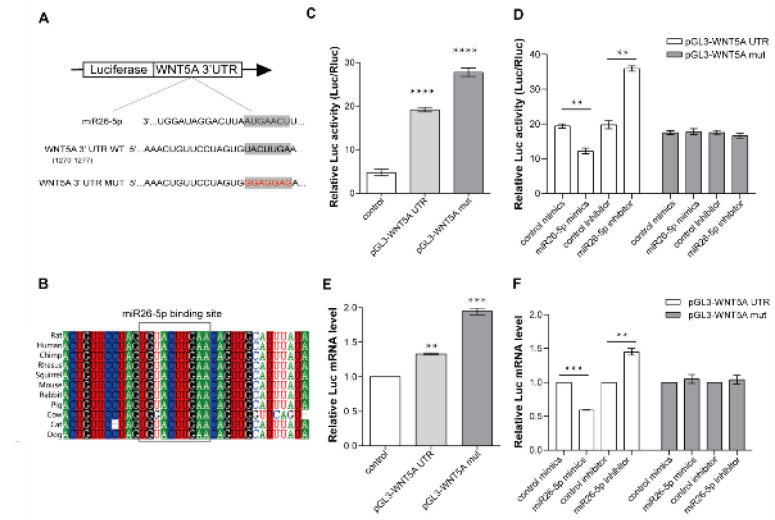
Identification of WNT5A as a direct target of miR26-5p

Next, qRT-PCR was used to detect miR26-5p level in the CBDL rat lung. The results revealed that miR26-5p expressed the highest in the lung tissue of normal rats, more than twice in that of the kidney (Figure 3A). In the CBDL rat lung, miR26-5p expression levels were reduced during the CBDL rat progression (Figure 3B). However, the expression level of WNT5A was opposite that of the miR26-5p (Figure 3C). And we found that there was a binding site of miR26-5p in 3’UTR of the WNT5A gene (Figure 4A). Furthermore, this binding site is very conversed across species (Figure 4B). The results indicated that miR26-5p may inhibit WNTN5A expression.

***MiR26-5p inhibits expression of WNT5A***

In order to analyze whether miR26-5p specifically binds to WNT5A 3’UTR target sequences to play a regulatory role, we constructed a luciferase reporting system. The result showed that pGL3-LUC-WNT5A UTR and pGL3-LUC-WNT5A UTR MUT transfected PMVECs showed higher Luc activity, and Luc mRNA transcription was also induced (Figure 4C). Then, miR26-5p specific mimic and inhibitor were used to treat PMVECs to achieve specific up-regulation or down-regulation of miR26-5p content in PMVECs (Figure 4D). Results showed that miR26-5p mimic specifically reduced LUC enzyme activity by two-thirds. The miR26-5p specific inhibitor enhanced LUC enzyme activity by two folds. However, control mimics and inhibitors can not change the Luc activity (Figure 4D). qRT-PCR results confirmed that the Luc gene transcript was in line with trend growth (Figure 4E, F). It indicated that miR26-5p is an important negative regulator of WNT5A by specially targeting WNT5A 3′ UTR. The result suggested that miR26-5p inhibits WNT5A expression directed by binding with the 3’UTR motif.

***MiR26-5p inhibits PMVEC proliferation by down-regulating the expression of WNT5A***

**Figure 5:**
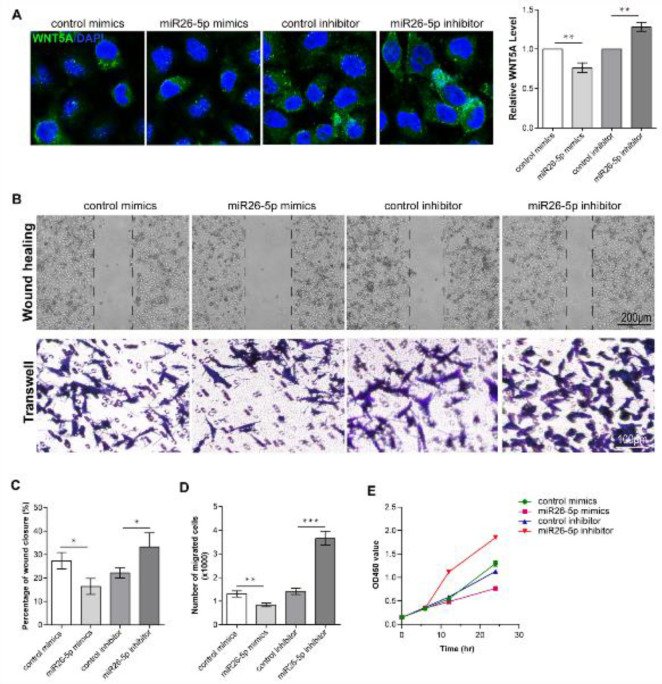
Identification of miR26-5p inhibition of PMVECs proliferation and migration

To identify miR26-5p regulation of PMVEC proliferation, we used miR26-5p specific mimics and inhibitor treatment of PMVECs. WNT5A staining showed that WNT5A expression was down-regulated when miR26-5p was overexpressed by mimic. But when miR26-5p was reduced by inhibitor, WNT5A expression was up-regulated (Figure 5A). Then, PMVEC proliferation and migration were assessed by CCK8 assay, transwell assay, and wound healing assay. The result of the CCK-8 assay showed that PMVECs proliferation was significantly inhibited by the treatment of miR26-5p mimic. The data showed that the effect of miR26-5p inhibitor on the proliferation of PMVECs was opposite to that of miR26-5p mimic (Figure 5E). After miR26-5p mimics were treated, PMVECs migration was inhibited significantly. Meanwhile, after the miR26-5p inhibitor was treated, PMVECs migration was induced significantly (Figure 5B-D). It suggested that miR26-5p affects PMVEC proliferation and migration by inhibiting WNT5A expression.

## Discussion

The clinical manifestations of HPS are pathologic pulmonary microvascular dilatation, obstructed gas exchange, and abnormal arterial oxygenation due to chronic liver disease and/or portal hypertension (25). It has been reported that the risk of death in HPS patients is more than twice that in patients without HPS cirrhotic. Persistent and severe dyspnea (PaO_2_ decreased by 5 mmHg per year) was considered to be the most representative symptom of HPS patients (26). The main pathologies of HPS include pathologic vascular dilatation, microvascular dysplasia, and hypoxemia. Up to now, studies on the pathophysiological mechanism related to HPS are still unclear. There is no specific intervention drug in clinical practice up to now, and liver replacement is the only option for this disease (27).

Our data showed that HPS rats had a significantly increased number of pulmonary micro-vessels, a higher lung injury score, and a significantly decreased PaO_2_ level, compared with that of the sham group. Immunohistochemistry and quantitative PCR results showed that the expression level of WNT5A in the lung tissues of HPS rats was significantly increased, in contrast to the sham group. WNT5A is not only produced primarily in the endothelium but also other tissues, like adipose. It has been reported that WNT5A regulates heart and lung development, and tumor progression (28-30). And WNT5A was involved in the angiogenesis of myocardial injury in diabetic mice and tumor development (29, 31). Our results confirm the relationship between WNT5A and the occurrence of pathological angiogenesis in HPS. Meanwhile, due to the important role of WNT5A in regulating pulmonary vascular permeability, it is reasonable to speculate that WNT5A participates in HPS-related pulmonary microvascular pathological angiogenesis.

Sequence-specific binding of miRNAs to their target messenger RNA (mRNAs) can inhibit mRNA translation or inhibit the action of nuclease *in vivo* to target mRNA (32), which has been widely accepted as a mechanism of action. Most microRNAs are located inside cells, except for some that circulate in body fluids. Therefore, in the different regulation levels of gene expression, miRNA-mediated gene regulation is one of the important mechanisms for gene post-transcription regulation. The statistics show that at least 30% of the genes in mammals are regulated by miRNA. This regulation plays a very important role in the development of various human diseases (33). In the HPS mouse model, miR-101 can inhibit the proliferation of PMVECs (34). The regulation of WNT5A on HPS-related IPVD pathological changes may be also related to miRNAs. Therefore, in this study, a miR26-5p binding site on WNT5A through bioinformatics analysis was identified. This suggests that the expression of WNT5A may be regulated by miR26-5p. Our data further confirm this hypothesis. The miR26-5p function has been established in cell proliferation, cell migration, and invasion. Down-regulation of miR26-5p is associated with breast cancer risk, but its role in HPS is unclear. However, the research on miR26-5p in HPS is still very little, and its function and mechanism of action have not been reported. In this study, we figured out the expression trend, function, and regulation mechanism of miR26-5p in HPS angiogenesis. Our results established that the expression level of miR26-5p in the CBDL rat lung was significantly lower than that of normal rats. The same changes were observed in the expression of miR26-5p in PMVECs treated with the CBDL rat serum. Therefore, it is reasonable to assume that miR26-5p may control the HPS process by regulating the WNT5A signaling pathway.

In addition, all of our data showed that miR26-5p acts as a negative regulator of WNT5A expression by western blot, luciferase reporting systems, and immunofluorescence. Since the abnormal proliferation of PMVECs is the most important pathological change of HPS-related pulmonary microvascular hyperplasia *in vitro*, Ki67 staining and CCK-8 method were used to analyze the regulation of miR26-5p on the PMVECs proliferation. It showed that miR26-5p mimics significantly inhibited the proliferation of PMVEC.

A) Histology assessment of lung pathology in the HPS rat model (n=20). B) Lung injury score of the lungs from CBDL rats or sham rats. C) Number of pulmonary microvascular in the lungs of CBDL rats. D, E) pO_2_ and pCO_2_ level in HPS rat. (compared with sham group; *P<0.05, **P<0.005, ***P<0.001, and ****P<0.0001)

A) Immunohistochemistry detected WNT5A expression in the lung of CBDL rats. B) qRT-PCR detected WNT5A gene transcription levels in the lungs of HPS rats. C) Western blot analysis of WNT5A protein synthesis in the lungs of HPS rats. D) Cck8 assay detected PMVEC proliferation by WNT5A overexpression and knockdown virus treatment. E, F) Wound healing and transwell assay were used to analyze PMVECs migration after virus treatment. G) IF staining of WNT5A. (A-C, Compared with sham group; D-G, compared with control; *P<0.05, **P<0.005, ***P<0.001, and ****P<0.0001)

A) qRT-PCR analysis miR26-5p level the tissues of normal rats. B) qRT-PCR analysis miR26-5p expression trend in the lung at different pathological stages of HPS. C) qRT-PCR analysis of WNT5A transcriptional level in lungs at different pathological stages of HPS. D) Western blot analysis of WNT5A protein synthesize after miR26-5p mimics or inhibitor-treated PMVECs. E) qRT-PCR analysis of WNT5A transcript after miR26-5p mimics or inhibitor-treated PMVECs. (B, C, compared with the sham group; D, E, compared with control mimics/control inhibitor; *P<0.05, **P<0.005, ***P<0.001, and ****P<0.0001)

**Table 1:** miR26-5p putative target genes predicted by online bioinformatics website

**Target gene**	**Transcript ID**	**Cumulative weighted context score**	**Total context score**	**Aggregate PCT**
HMGA2 (high mobility group AT-hook 2)	ENST00000403681.2	-0.85	-0.85	0.89
PTEN (phosphatase and tensin homolog)	ENST00000371953.3	-0.76	-0.91	> 0.99
ZDHHC20 (zinc finger, DHHC-type containing 20)	ENST00000320220.9	-0.68	-0.8	0.96
ZDHHC6 (zinc finger, DHHC-type containing 6)	ENST00000369405.3	-0.68	-0.68	0.79
ART3 (ADP-ribosyltransferase 3)	ENST00000341029.5	-0.68	-0.68	< 0.1
ZNF259 (zinc finger protein 259)	ENST00000227322.3	-0.34	-0.44	0.42
HOXD13 (homeobox D13)	ENST00000392539.3	-0.33	-0.34	0.74
SRCAP (Snf2-related CREBBP activator protein)	ENST00000262518.4	-0.33	-0.33	0.98
ZNF462 (zinc finger protein 462)	ENST00000277225.5	-0.3	-0.32	0.94
ZNF385B (zinc finger protein 385B)	ENST00000410066.1	-0.28	-0.28	0.69
RAB3IP (RAB3A interacting protein)	ENST00000483530.2	-0.28	-0.31	0.41
DCBLD1 (discoidin, CUB, and LCCL domain containing 1)	ENST00000338728.5	-0.28	-0.3	0.39
LMLN (leishmanolysin-like metallopeptidase M8 family)	ENST00000330198.4	-0.28	-0.28	0.94
AL355390.1 (Uncharacterized protein)	ENST00000325811.1	-0.27	-0.27	0.34
WNT5A (wingless-type MMTV integration site family, member 5A)	ENST00000474267.1	-0.27	-0.34	< 0.1
METAP2 (methionyl aminopeptidase 2)	ENST00000323666.5	-0.27	-0.34	0.68
DDR2 (discoidin domain receptor tyrosine kinase 2)	ENST00000367922.3	-0.27	-0.27	0.67
ALDH5A1 (aldehyde dehydrogenase 5 family, member A1)	ENST00000357578.3	-0.27	-0.51	0.79
CEP350 (centrosomal protein 350kDa)	ENST00000367607.3	-0.26	-0.31	0.94
PHF21A (PHD finger protein 21A)	ENST00000257821.4	-0.26	-0.28	0.94
HOMER1 (Homer homolog 1)	ENST00000334082.6	-0.26	-0.27	0.67

A) Binding site of miR26-5p in the 3’UTR of WNT5A. B) miR26-5p binding site is highly conserved across species. C) Luciferase activity was analyzed after PMVECs were co-transfected with pGL3-WNT5A 3’UTR or pGL3-WNT5A mut. D) Luciferase activity was analyzed by treatment of miR26-5p mimics or inhibitors after co-transfection with pGL3-WNT5A 3’UTR or pGL3-WNT5A mut plasmid. E) qRT-PCR detected LUC gene expression by co-transfection with pGL3-WNT5A 3’UTR or pGL3-WNT5A mut. F) qRT-PCR detected LUC gene expression by treatment of miR26-5p mimics or inhibitors after co-transfection with pGL3-WNT5A 3’UTR or pGL3-WNT5A mut plasmid

A) Immunofluorescence staining analyzed WNT5A expression after PMVECs transfected with miR26-5p mimics or inhibitors. B) Wound healing and transwell assay were used to analyze PMVECs migration. C) Percentage of wound closure statistics of wound healing assay. D) migrated cells statistics of transwell assay. E) OD450 values detected by CCK8 assay

## Conclusion

Our research confirmed the occurrence of pulmonary microvascular hyperplasia in HPS. The expression of WNT5A was significantly increased in CBDL rat lungs, while the expression of miR26-5p was significantly inhibited. The expression trend of miR26-5 in PMVECs treated with serum from CBDL rats also showed the same change. Reduced proliferation of PMVEC was observed after transfection with miR26-5p mimics. However, the regulation of miR26-5p on the proliferation of PMVECs is realized through WNT5A, because the change of WNT5A expression directly affects the proliferation of PMVECs. This study showed that miR26-5p, WNT5A, and PMVECs were directly involved in pulmonary microvascular hyperplasia in HPS rats. Our findings demonstrate that microRNAs may be a potential tool for novel HPS therapeutic strategies. Nevertheless, in the next step, we had to construct miR26-5p knockout and overexpression animal models to further verify our findings. In the short run, understanding the abnormal PMVEC cell proliferation mechanisms will provide a basis for inhibiting pathological micro angiogenesis and creating useful targeted therapies for HPS.

## Authors’ Contributions

JC and JQW designed the experiments; JC, FG, and DL performed experiments and collected data; JC and JQW discussed the results and strategy; JQW supervised, directed, and managed the study; JC, FG, DL, and JQW approved the final version to be published.

## Conflicts of Interest

The authors declare no conflicts of interest.
